# Eliminating Human African Trypanosomiasis: Where Do We Stand and What Comes Next>

**DOI:** 10.1371/journal.pmed.0050055

**Published:** 2008-02-26

**Authors:** Pere P Simarro, Jean Jannin, Pierre Cattand

## Abstract

While the number of new detected cases of HAT is falling, say the authors, sleeping sickness could suffer the "punishment of success," receiving lower priority by public and private health institutions.

In the early part of the twentieth century, human African trypanosomiasis (HAT), also known as sleeping sickness, decimated the population in many parts of sub-Saharan Africa. In the 1930s, the colonial administrations, conscious of the negative impact of the disease on its territories, established disease control programmes. Systematic screening, treatment, and follow-up of millions of individuals in the whole continent led to transmission coming to a near halt by the 1960s.

With the advent of independence in most countries where HAT was endemic, the newly independent authorities had other priorities to deal with. The rarity of HAT cases, and a decline in awareness of how the disease could return, led to a lack of interest in disease surveillance. Over time the disease slowly returned, and some thirty years later, flare-ups were observed throughout past endemic areas (Figure 1).

**Figure 1:**
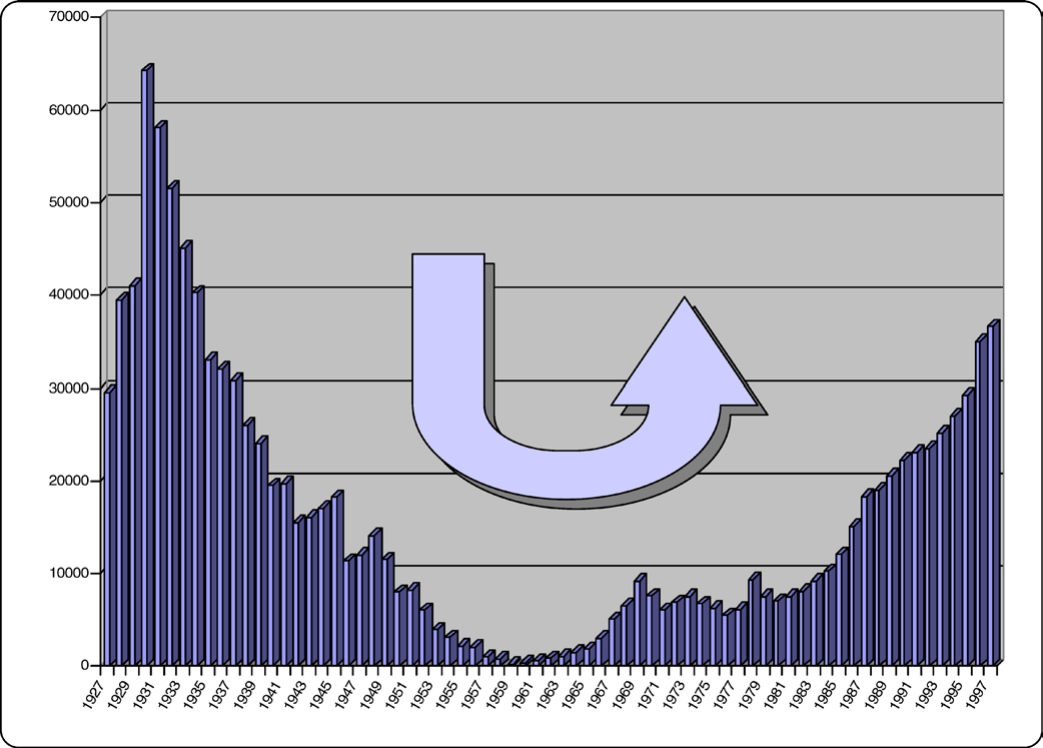
New Cases of Sleeping Sickness Reported for All Africa between 1927 and 1997

Since 1995, the World Health Organization (WHO) has on many occasions expressed its concern about the rise in HAT cases. The World Health Assembly has passed several resolutions in an attempt to stem this rise. However, social upheavals, wars, and population movements, combined with lack of awareness and shortage of funds, prevented any progress in interrupting transmission, and the disease continued to evolve and spread.

In a 1997 resolution, WHO strongly advocated access to diagnosis and treatment and the reinforcement of surveillance and control activities, concurrently setting up a network to strengthen coordination among all those actively concerned by the problem [1]. As a consequence, the public and private sector granted stronger support to HAT surveillance, control, and research.

## Pathology, Clinical Features, and Epidemiology

**Figure 2:**
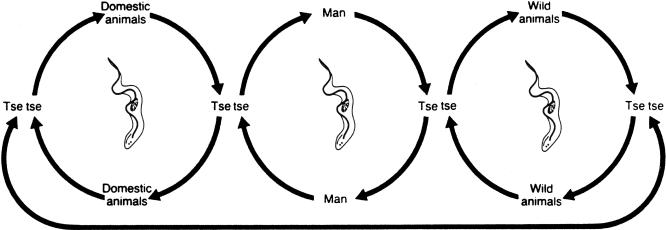
HAT Transmission Cycle In T. b. gambiense the cycle is mostly human-to-human (central circle); occasionally transmission may occur from animal to human. In T. b. rhodesiense the animal reservoir plays an important role in the cycle, thus sustaining parasite transmission and human infections.

HAT is a vector-borne parasitic disease that is fatal if left untreated. It is caused by a single-celled protozoa belonging to the Trypanosoma genus. Parasites are transmitted to humans by the bite of a tsetse fly (Glossina genus) that has acquired the infection from human beings or from animals harbouring the human pathogenic parasites (Figure 2).

Tsetse flies, and subsequently sleeping sickness, are usually found in remote sub-Saharan rural areas where health systems are weak or non-existent. For reasons that are so far unexplained, there are many regions where tsetse flies are found but sleeping sickness is not. Sleeping sickness, coupled with nagana, the animal form of African trypanosomiasis, has been a major obstacle to sub-Saharan African rural development and a stumbling block to agricultural production. On the one hand, human infections reduce labour resources, while on the other, the animal disease limits availability of meat and milk and deprives African farmers of draught animal power, substantially minimising crop production. Therefore, both human and animal trypanosomiasis are implicated in the underdevelopment of the African continent, and are considered a major obstacle in the establishment of a flourishing agriculture to provide food security and to lead to sustainable economic growth and healthy populations.

The rural populations that live in regions where transmission occurs and depend on agriculture, fishing, animal husbandry, or hunting are the most exposed to the bite of the tsetse fly and therefore to the disease. Displacement of populations, war, and poverty are important factors leading to increased transmission. The disease develops in areas whose size can range from a village to an entire region. Within a given area, the intensity of the disease can vary from one village to the next.

The human disease takes two forms, depending on the parasite involved. Trypanosoma brucei gambiense is found in west and central Africa. This form represents more than 90% of reported cases of sleeping sickness, and causes a chronic infection. A person can be infected for months or even years without major signs or symptoms of the disease. When symptoms do emerge—such as severe headaches, sustained fever, sleep disturbances, alteration of mental state, and neurological disorders—the patient is often already in an advanced disease stage where the central nervous system is affected. Trypanosoma brucei rhodesiense is found in eastern and southern Africa. This form represents less than 10% of reported cases, and causes an acute infection. The first signs and symptoms—such as chancre, occasional headaches, irregular fevers, pruritus, and the development of adenopathies—are observed after a few weeks or months. Following this first stage, when the parasite has invaded the blood and lymph subsequent to the infective bite of the fly, the disease develops rapidly into a second stage when parasites cross the blood–brain barrier, invading the central nervous system.

**Figure 3:**
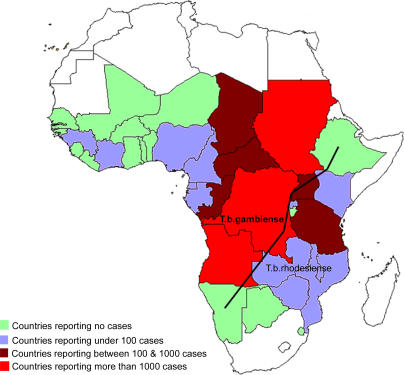
Map of Africa Showing the Epidemiological Status of Countries Considered Endemic for the Disease

Thirty-six sub-Saharan countries are considered endemic for one or the other form of the disease, despite the fact that some of them have reported no cases in the last decade (Figure 3).

## Current Situation

Since WHO expressed its concerns in 1995, there have been great improvements in HAT control. In addition to political will at the highest levels, capacities for control and surveillance in endemic countries were strengthened through training and the provision of equipment for screening, diagnosis, and treatment.

The abatement of social upheavals and civil wars in many countries where HAT was endemic facilitated access to diagnosis and treatment, which was then enhanced by the financial and technical support from WHO for outreach activities, and by securing production and free distribution of drugs. Between 1995 and 2006, the total number of new cases reported was reduced by 68%.

With WHO support and commitment, it became possible to reinforce exhaustive screening of the population at risk, using a combination of immunological and parasitological tests. The card agglutination test for trypanosomiasis (CATT) developed in 1978 is widely used for screening the population affected by T. b. gambiense but is not applicable in T. b. rhodesiense areas. CATT-positive results are not sufficiently sensitive and specific to establish a definitive diagnosis, and therefore parasitological tests must be performed to confirm the presence of parasites in seropositive individuals. Such tests consist of microscopic examination of the lymph and blood. They are considered cumbersome and insufficiently sensitive to ascertain absence of infection [2]. Diagnosis is followed by systematic stage determination, which consists of assessing the cerebrospinal fluid for white blood cell increases, elevated protein concentrations, and the presence of parasite—thus requiring a lumbar puncture, an invasive procedure that is not well accepted by patients.

Treatment today relies on four parenteral drugs: suramin for first-stage rhodesiense pentamidine for first-stage gambiense melarsoprol for the second stage of both forms of the disease, and eflornithine, which is only effective in the second stage of the gambiense form. The management of patients using any of these drugs is cumbersome and risky, requiring well-trained staff.

Despite success in reducing the number of cases reported, the complexity of the current tools available to control the disease does not allow the full involvement of the health care system, hampering the sustainability of HAT surveillance and control as discussed below.

### Epidemiological update.

In early 2006, WHO published an update on the disease situation and ongoing control activities in each of the 36 countries considered endemic for HAT [3]. The two forms of the disease were considered separately, due to their different epidemiological characteristics.

**Figure 4:**
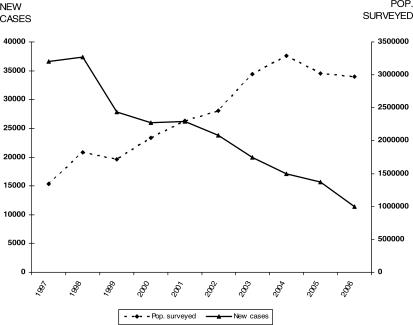
T. b. gambiense: Comparative Evolution Curves between Population Placed Under Active Surveillance and New Cases Reported (1997–2006)

Between 1997 and 2006, the gambiense form (97% of the total cases reported at continental level) responded well to intensive control activities mainly focused on the human reservoir (the animal reservoir was considered to have only a minor impact on the transmission process). The number of people under active surveillance increased, and the number of new cases decreased (Table 1 and Figure 4). However, control activities focusing on the human T. b. rhodesiense reservoir (3% of the total number of cases reported) were found insufficient to control the disease, probably due to the role played by the animal reservoir on transmission. Thus, T. b. rhodesiense showed only a small decrease in the number of cases (Table 2).

**Table 1:**
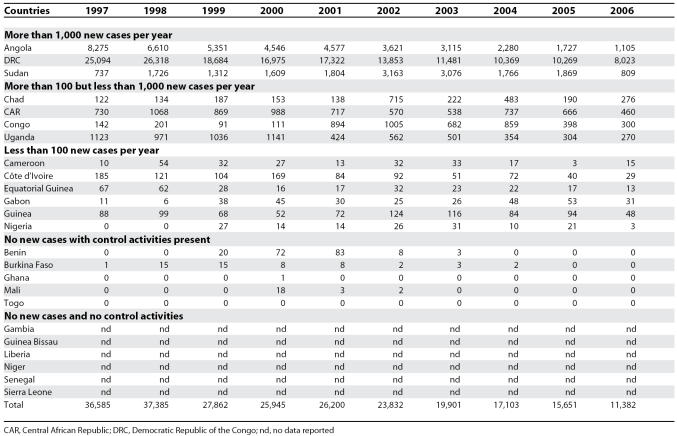
T. b. gambiense Sleeping Sickness: New Cases Reported between 1997 and 2006

**Table 2:**
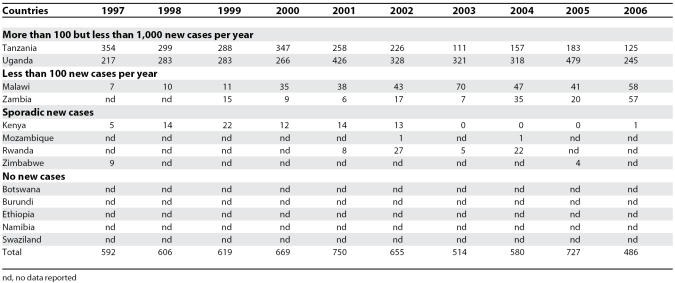
T. b. rhodesiense Sleeping Sickness: New Cases Reported between 1997 and 2006

Out of 36 countries considered endemic for HAT, 24 are experiencing T. b. gambiense transmission. In these countries, there was a 69% reduction in the number of new cases reported during the period from 1997–2006. In 2006, 11 out of the 24 countries reported no cases; six of them had no control activities and reported no cases over a decade; and the other five implementing control activities reported only sporadic cases during the 1997–2004 period. Together, the cases in these 11 countries represent only 0.1% of the total gambiense cases reported.

Six countries reported an average of less than 100 new cases per year, representing 1.2% of the total gambiense cases. All these countries (except Nigeria) have National Sleeping Sickness Control Programmes and carry out regular control activities. Four countries, with well-established Control Programmes and regular control activities, have reported more than 100 but less than 1,000 new cases per year, representing 8.8% of the total gambiense cases reported. Finally, three countries have reported an average of more than 1,000 new cases per year during the period from 1997–2006; together they represent 89.9% of the total gambiense cases reported (Table 1).

In the 13 countries endemic for rhodesiense there was a 21% reduction in the number of new cases reported during the 1997–2006 period (Table 2). However, only Kenya, Malawi, Tanzania, and Uganda implemented control activities (since Uganda is affected by both forms of the disease, the country appears in Tables 1 and 2). Out of these 13 countries, five reported no cases over a decade and four reported sporadic cases. Together, these four countries reporting only sporadic cases represent 2.5% of the total rhodesiense cases reported during the 1997–2006 period. Two countries reported an average of less than 100 new cases per year, representing 8.7% of the total rhodesiense cases, and two reported more than 100 but less than 1,000 new cases per year, representing 88.8% of the rhodesiense cases reported.

### The road to elimination.

To achieve the 1997 World Health Assembly elimination resolution [1], the WHO HAT Surveillance and Control Programme established a new initiative based on a global alliance bringing together all actors concerned about the disease [4]. In 2003, the World Health Assembly called on member states to sustain the effort to eliminate the disease as a public health problem, which led the WHO programme to intensify its coordinating efforts, bringing together national control programmes, nongovernmental organisations, research institutions, and other concerned United Nations Agencies (under the Programme against African Trypanosomiasis, PAAT) [5], as well as private and public contributors (Sanofi-Aventis, Bayer HealthCare, the Bill & Melinda Gates Foundation, and the Belgium and French Cooperation). With this broad coalition, field activities were scaled up, leading to better knowledge of the disease distribution and a reduction in new cases by 2006, as described above. The current prevalence and incidence figures are believed to reflect the overall situation quite accurately, in contrast with the uncertainties surrounding the figures prior to 1997.

Given that in 2006, 20 out of 36 endemic countries achieved or were close to achieving the target of reporting no new cases, and eight countries reported less than 100 new cases per year, elimination has become a feasible objective in many countries endemic for HAT. With elimination in mind, in May 2007 WHO organised an Informal Consultation on Sustainable Sleeping Sickness Control, during which endemic country representatives debated the current disease landscape and concluded that elimination was possible.

### Political will.

During the July 2000 Organization of African Unity (now the African Union) summit held in Lomé, Togo, the African Heads of State and Government adopted the decision to collectively embark on a Pan African Tsetse and Trypanosomosis Eradication Campaign (PATTEC). This campaign was based on the realisation that (1) solving the tsetse fly and disease problem would be an important contribution to Africa's development, and (2) this could not be done by a single country acting alone. A task force of African experts concluded that such a campaign was not only technically feasible, but economically productive [6].

Implementation is on its way; six countries have recently received financial support from the African Development Bank and have initiated the first phase of a PATTEC project. In addition, four countries in the Kwando/Zambezi region have begun PATTEC activities, with very encouraging results.

## The Next Steps

### Integration of activities.

The challenge for the immediate future is to avoid repeating past mistakes, and to achieve cost-effective, sustainable HAT surveillance and control. Sustainability can only be achieved through an integration of activities in a strengthened health system able to face such responsibilities. The current approach should include specialised teams *and* health care systems, rather than falling back on the former debate between the value of specialised teams *or* primary health care. In other words, specialised teams and primary health care need to work together synergistically [7].

But integration is not a simple delegation process. Major responsibilities cannot simply be passed on to the existing health services of remote rural areas inappropriately trained and equipped to handle HAT control. Integration must mean the active participation of a strengthened health system capable of implementing surveillance and control activities, buttressed by specialised HAT national staff. Unfortunately, the existing tools limit the full participation of the health care system staff in controlling the disease. The two main technical bottlenecks are the lack of a sensitive and specific diagnostic test and of a new drug that is cheap, safe, and easy to administer.

### New approaches to surveillance and control.

To sustain recent achievements in HAT control and the epidemiological downward trend, it will be necessary to develop a novel approach for surveillance and control adapted to the new requirements. This approach consists of an integration process involving national health care systems. Implementation, however, will require better tools than those presently available for diagnosis and treatment. Such a health systems–based approach may be adequate for areas affected by T. b. gambiense but in areas affected by T. b. rhodesiense disease control cannot rely exclusively on human health services and will have to involve veterinary and entomological services as well.

### Developing new diagnostic tools.

Attempts to identify new antigens should result in more specific and sensitive tests for serodiagnosis of the disease, while changes in test format (i.e., the development of non-invasive saliva tests [8]) should result in more user-friendly tests. Much progress has been made in the development of molecular tools. Specific genes for both T. b. gambiense and T. b. rhodesiense have been identified [9–11] for PCR-based detection of infection. Molecular dipstick tests allow easier reading of the PCR result [12], and the first results using loop-mediated isothermal amplification [13] are encouraging.

Disease bio-markers are being investigated using proteomics, such as surface-enhanced laser desorption/ionisation time-of-flight mass spectrometry (SELDI-ToF-MS) [14]. However, these newly developed techniques, claimed to be substantially more sensitive and specific than those available in the field today, often rely on complicated equipment. As a result, the test protocols are not compatible with prevailing conditions at HAT treatment centres in rural Africa. WHO has established a collaboration with the Foundation for Innovative New Diagnostics (http://www.finddiagnostics.org/) to develop new simple diagnostic tools for the control of HAT that meet the requirements of a sustainable elimination approach. The desired characteristics of a new test were defined as being “ready for use”, stable at room temperature, and affordable by national health systems. The new test should provide an uncontroversial diagnosis of both forms of the disease and require minimum training and equipment to allow its execution by any health worker.

### Developing new tools for determining stage of disease.

As long as there is no safe and effective drug available to treat both stages of the disease, determining disease stage will remain necessary. Some progress has been made through the development of a point-of-care card agglutination test for immunoglobulin M quantification in cerebrospinal fluid [15]. Although this test appears highly promising in establishing central nervous system involvement, its accuracy and feasibility in the field still need to be ascertained. The study of anti-neurofilament and anti-galactocerebrosides antibodies [16] may open new avenues for staging the disease. Unfortunately, all these techniques continue to require a lumbar puncture. Stage markers in other body fluids such as serum, urine, or saliva could become ideal tests to avoid the invasive procedure of a lumbar puncture, but remain to be identified.

Another possible technique for the diagnosis of central nervous system involvement is the measurement of sleep-onset rapid eye movement by polysomnography, a method that involves assessing the sleep pattern of patients [17]. However, although it is not invasive, polysomnography has not yet been proven to be universally accurate. Obviously, much work still needs to be done to make improved staging tests available to health workers in endemic areas.

### Advances in drug development.

Eflornithine was developed over 20 years ago, and was registered for the treatment of gambiense disease in 1990. While the drug is safer than melarsoprol [18], eflornithine does have side effects: fever, unusual bleeding and weakness, diarrhoea, nausea, stomach pain, and vomiting are common, while rarer side effects such as convulsions, loss of hearing, hair loss, headache, anaemia, leucopenia, and thrombocytopenia have also been observed [19]. The administration of eflornithine, which requires multiple daily infusions, limits its use in the context of rural Africa, despite the determination of some programmes to use it as a first-line drug.

Recently a short-course melarsoprol treatment was developed [20,21]. Unfortunately, it does not provide a safer treatment; however, it has substantially reduced the hospitalisation time of patients and as a consequence the cost of treatment.

With the development of parasite resistance to some of the available drugs [22], a number of studies have attempted to combine existing drugs to overcome treatment failures [23,24]. A clinical trial, sponsored by Médecins sans Frontières-Holland, the Drugs for Neglected Diseases Initiative, and the UNICEF-UNDP-World Bank-WHO Special Programme for Research and Training in Tropical Diseases, is currently ongoing to test a combination of eflornithine and nifurtimox, the latter being a drug registered for the treatment of American trypanosomiasis (Chagas disease). The aim of the study is not only to improve efficacy and simplify administration, which would contribute to easier field use and reduce cost, but also to find a way to avoid the development of resistant strains to eflornithine. Such combination therapy remains far from ideal, since it continues to require intravenous administration with costly and complicated logistics and skilled staff. Furthermore this combination will only be effective for T. b. gambiense and would certainly not be safe enough to be used in first-stage patients; thus it will not help in avoiding the risky staging process.

A new oral drug called DB289 is in the final clinical trial phase. Given its oral administration, it should be considered an important step forward. Unfortunately, the drug requires ten days of treatment, twice a day, and is only effective in the first stage. Consequently, DB289 cannot be considered a new drug that would be a major advance in control of HAT.

The major challenge in developing a new drug that can ensure sustainable disease control will be to find a safe and affordable, orally administered drug that is effective against both forms of the disease, in both disease stages, and that does not require any particular skills or care to administer. The ideal regimen should not last more than a few days, thus making it manageable by peripheral health staff in an out-patient context.

### Advances in vector control.

Current vector control interventions involve the use of insecticides (through the sequential aerosol spraying technique, insecticide-treated targets [25], or insecticide-treated animals [26,27]); the use of traps [28,29]; and the sterile insect technique (SIT) [30].

The sequential aerosol technique, which uses extremely low concentration of insecticide through several consecutive aerial sprayings, can effectively clear large areas of tsetse flies in a relatively short time, but it is expensive and requires substantial economic and infrastructure support. Pourons or selective spraying application of insecticides to animals on which tsetse feed are another effective means of vector control.

Odour-baited targets or traps have been used in many countries to effectively suppress tsetse population. The relative low cost and simplicity of the traps or targets recommends them for use by local communities, but they are applied on a scale so small that control efforts are bound to be frustrated by re-invasion.

While effective baits have been developed for savannah tsetse, to date no such baits exist for riverine tsetse, which are major vectors of HAT. However, research continues in an attempt to develop effective baits for the latter species.

The SIT, which involves the release of laboratory-reared and sterilised males to compete with wild males so that females inseminated by them produce no offspring, has been effectively used for eradication of tsetse (G. austeni), for example, in Unguja Island in Zanzibar [30]. The cost of SIT is, however, exorbitant. The feasibility of this costly approach in areas where multiple species are present remains doubtful [31].

The recent availability of genomics of tsetse-symbiotic bacteria [32] is of interest since in the absence of their gut flora, tsetse flies are severely impaired in their longevity and reproduction. Two bacteria have been implicated in modifying vector competence of their host, and a third symbiont can confer mating sterility. However, further research is needed to turn such new knowledge into practical use for disease control.

Despite the considerable progress made in controlling the vector, an ideal methodology easily accessible to the population at risk still does not exist.

## Conclusion

While the number of new detected cases of HAT is falling, sleeping sickness could suffer the “punishment of success,” receiving lower priority by public and private health institutions with the consequent risk of losing the capacity to maintain disease control. While waiting for new tools for sleeping sickness control, the greatest challenge for the coming years will be to increase and sustain the current control efforts using existing tools. Effective surveillance and control followed by good reporting will be crucial. Furthermore, advocacy in endemic countries should continue to be maintained in the face of decreasing cases reported; sleeping sickness should retain its high priority with health policy makers and planners. Research must be encouraged to resolve the technical issues preventing the development of a new approach to surveillance and control that could be sustained by countries themselves.

Since elimination of the disease has been considered feasible, WHO will adopt the conclusions of countries where HAT is endemic, who have demonstrated that: (1) the participation of existing health systems is not only desirable but essential for surveillance and control sustainability; (2) the development of new diagnostic tools and drugs is crucial to guarantee the effective participation of existing health structures; and (3) the maintenance of a specialised central structure at national level is necessary to ensure the coordination and overall technical assistance needed. In that context, WHO is ready to take up the challenge and continue to lead countries, supporting and coordinating the work of all the actors involved.

## Abbreviations

CATT: card agglutination test for trypanosomiasis
HAT: human African trypanosomiasis
PATTEC: Pan African Tsetse and Trypanosomosis Eradication Campaign
SIT: sterile insect technique
WHO: World Health Organization
